# New Jersey’s Opportunity

**Published:** 1900-12

**Authors:** 


### NEW JERSEY'S OPPORTUNITY.

WITH New York upon one side and Pennsylvania upon the other, the "Garden State" has had an exhibition of unexcelled progress in veterinary association work. Not lacking in numbers nor in individual merit of her veterinary representatives, that State has wasted her powers in the past because there lacked a single leader among her numbers who could create a common worshipping ground where all her various devotees of the profession might erect a common altar of veterinary progress before which all might worship. There is a strange fascination innate in the average man that he must con the same pages as others, learn the same lessons by personal experiences, and ever be forgetful of history repeating itself. So with New Jersey's profession; she had to learn through her own trying experiences that "united, we stand; divided, we fall." She has learned well the lesson, she has lost much by the experience; but her ranks are now closed up, and there will be a common enemy to fight every barrier to veterinary progress and to wipe away every stumbling-block to future good work in higher veterinary education and better association work. Veterinary sanitary legislation and every field of veterinary advancement will feel the impulse of a strong force from her many able veterinary leaders. She will demonstrate at Atlantic City in September her strength and power. She will place in the forefront her wisest leaders, and she will grasp the one lacking opportunity to make her armor complete in her future battles in legislative halls, in municipal councils, in animal industry organizations, until there will mark a progress for the welfare of her people second to none in the United States. Let the Association meet in goodly numbers along her wonderful seashore front and give the needed impulse in this centre, such as has followed in the South, the West, the Northwest, and every other centre where the A. V. M. A. has assembled, and let New Jersey seize in the fullest sense all the good that flows therefrom. The Lowes, Dixons, Millers, Hawk, Lockwood, and many others equally well known as representative veterinarians of that State will fail in the sight of their confrères in sister States if they do not make this an epoch-making event in veterinary history in New Jersey. Let New York, Pennsylvania, and Maryland aid their colleagues in New Jersey, and the Atlantic City meeting will be a bright star in the galaxy of meetings that have crowned veterinary progress in the East with laurels worthy of the cause.

